# Human beta-defensin 3 contributes to the carcinogenesis of cervical cancer via activation of NF-κB signaling

**DOI:** 10.18632/oncotarget.12426

**Published:** 2016-10-04

**Authors:** Dan Xu, Bing Zhang, Chongbing Liao, Wei Zhang, Weijia Wang, Ying Chang, Yongping Shao

**Affiliations:** ^1^ Key Laboratory of Biomedical Information Engineering of the Ministry of Education, Department of Biological Science and Engineering, School of Life Science and Technology, Xi'an Jiaotong University, Xi'an, Shaanxi, China; ^2^ Center for Translational Medicine, Frontier Institute of Science and Technology, Xi'an Jiaotong University, Xi'an, Shaanxi, China

**Keywords:** human beta-defensin 3, cervical cancer, carcinogenesis

## Abstract

Human beta-defensin 3 (hBD3), an antimicrobial peptide (AMP) expressed in epithelium in response to various stimulations including human papillomavirus infection, has recently been found to be overexpressed in head and neck cancers and exhibit tumorigenic activities. However, the role of hBD3 in cervical cancer remains to be investigated. In this study, we showed by immunohistochemistry that hBD3 expression was elevated in cervical cancer samples of different stages versus the normal tissue, and was positively correlated with the progression of the disease. Overexpression of hBD3 in cervical cancer cell lines promoted cell proliferation by accelerating G1/S progression and enhanced cell migration and invasion *in vitro*. These oncogenic effects of hBD3 were associated with activation of NF-κB signaling. Using a mouse xenograft model, we further demonstrated that hBD3 overexpression promoted the growth of cervical cancer cells *in vivo*. Our results suggested that hBD3 is involved in the carcinogenesis and development of cervical cancer, and may serve as a biomarker or therapeutic target of this disease.

## INTRODUCTION

Human β defensins (hBDs) are small cationic peptides consisting of 30-50 amino acids with broad antimicrobial activity [1–3]. Different from their α siblings which are mainly expressed in neutrophils and intestinal Paneth cells, human β defensins are expressed in epithelial cells in response to various pro-inflammation signals and microbial invasion [2, 4–7].

Human β defensin 3 (hBD3) is a member of the β defensin family which is encoded by the gene DEFB103A [8, 9]. Besides the antimicrobial activity, hBD3 was recently found to play oncogenic roles in human cancers [10–13]. Overexpression of hBD3 was observed in oral squamous cell carcinomas (OSCC) and Kaposi's sarcoma and was implicated in the pathogenesis of these malignances [13–16]. Exogenous hBD3 accelerated the growth of OSCC cells and further stimulated the endogenous expression of hBD3 of these tumor cells [12]. HEK293 cells with overexpression of hBD3 showed significant increased tumor formation capacity and much improved tumor growth rate in nude mice [17]. Moreover, hBD3 in oral tumor microenvironment could traffick CCR2 positive tumor-associated macrophages (TAM), which are known to generate an inflammatory tumor microenvironment and promote tumor aggressiveness [16, 17]. Mburu et al. demonstrated that hBD3 induced CCR7 expression in primary squamous cell carcinoma of head and neck (SCCHN) tumor cells and stimulated the migration of tumor cells though activating the NF-κB pathway [16]. Furthermore, hBD3 conferred anti-apoptotic benefits to SCCHN cells by activating the PI3K/AKT signaling [16]. All these findings provide strong evidence for the oncogenic role of hBD-3 in oral squamous cell cancer. However, the potential oncogenic role of hBD3 in other cancer types were much less investigated.

Cervical cancer is the third leading malignancy of women worldwide [18]. Although infection by high-risk human papillomaviruses (HPV) has been proven a major determinant for cervical cancer [19, 20], whether the human immune mediators such as hBD3 also participate in the cervical carcinogenesis remains to be elucidated. Intriguingly, a very recent study by Dasgupta et al showed that hBD3 was under the regulation of the HPV oncoprotein E6 via the tumor suppressor p53 in HPV-infected cancer cells [21]. This finding provided a possible explanation for the high hBD3 expression associated with oral squamous cell carcinomas, some of which are caused by HPV infection.

Since cervical cancer is more directly linked to HPV infection, we hypothesized that hBD3 might also act as a tumor-promoting factor for cervical carcinogenesis. In this study, we examined hBD3 expression in cervical cancer patient samples of different disease stages and analyzed its correlation with disease progression. We also investigated the biological function of hBD3 by overexpression approach. Our results showed that hBD3 expression positively correlates with the progression of cervical carcinogenesis and that hBD3 contributes to the carcinogenesis of cervical cancer by activating the NF-κB signaling.

## RESULTS

### Upregulation of hBD3 expression in cervical carcinoma

**Figure 1:**
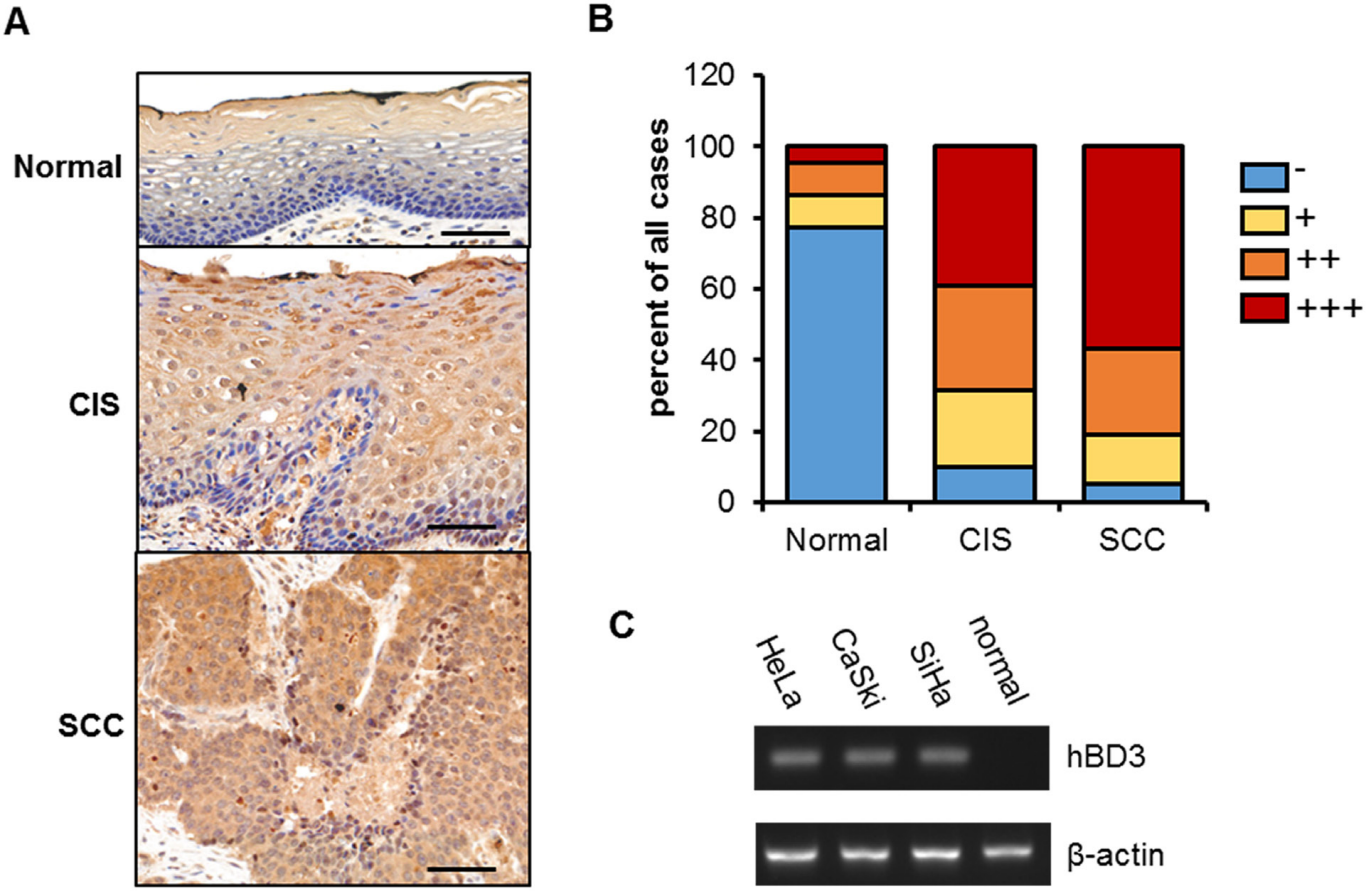
hBD3 expression in cervical cancer tissues and cell lines **A.** immunohistochemical staining showing hBD3 expression in normal cervical tissue (n=22), cancer in situ (CIS, n=41), and squamous cervical cancer (SCC, n=37), scale bar, 50 μm. **B.** Semiquantitative analysis of hBD3 staining. **C.** Quantitative RT-PCR analysis of hBD3 expression in normal cervix tissue and three cervical cancer lines: HeLa, CaSki and SiHa with β-actin being an internal control.

**Figure 2:**
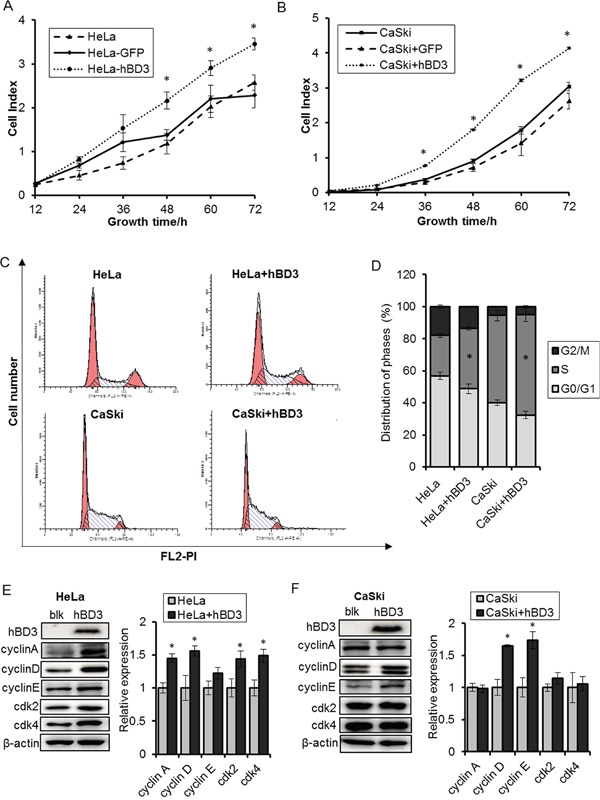
Effects of overexpression of hBD3 on cell proliferation hBD3 and eGFP (as control) was overexpressed in HeLa and CaSki cells by lenti-virus transduction. **A-B.** Real-time cell growth was monitored by the xCELLigence RTCA system for 72 hours. **C-D.** The cell cycle regulator profiles were analyzed 20h after seeding by flow cytometry. **E-F.** Cell cycle regulators were analyzed by western blot after hBD3 overexpression with β-actin being an internal control. All the quantitative data were presented as mean±SEM (n=3), *P<0.05.

To test whether hBD3 is associated with the carcinogenesis of cervical cancer, we performed immunohistochemistry studies on hBD3 in tissue samples from patients of different cervical cancer stages, including normal cervix (22 cases), carcinoma in situ (CIS, 41 cases) and cervical cancer (SCC, 37 cases) (Figure 1A). Both cytoplasmic and nuclear staining of hBD3 was observed in tissue samples. The positive hBD3 expression rates were 23% (5/22) in normal cervix, 90% (37/41) in cancer in situ, and 94% (35/37) in cervical cancers (Figure 1B, p<0.01) (Figure 1B). Moreover, analysis of the immunoreactivity score of hBD3 revealed that hBD3 expression levels correlated well with the progression of the disease. In contrast to the strong hBD3 staining in the cervical cancer tissues, hBD3 was not detectable in three cervical cancer cell lines (HeLa, CaSki and SiHa) using an antibody that reliably recognized the exogenous hBD3 either by western blot or immunofluoresence (Figure 2E–2F and Supplementary Figure S1), although the hBD3 mRNA levels were elevated in three cell lines versus the normal human cervical tissue (Figure 1C). This differential expression of hBD3 between tissue samples and cell lines suggested that the tumor microenvironment may participate in the regulation of hBD3 expression. Nevertheless, the low expression level of hBD3 in cervical cancer cell lines provided a good rationale for our subsequent overexpression studies.

### hBD3 promotes cervical cancer cell growth via regulation of the G1/S progression

Since hBD3 expression is elevated in cervical cancer samples and correlates with disease stages, we speculated that hBD3 may contribute to the cervical carcinogenesis. Given the low endogenous level of hBD3, we tested the impact of hBD3 on the growth of cervical cancer cells by an overexpression approach. HeLa and CaSki cell lines that stably express hBD3 or eGFP (as negative control) were established by lenti-viral transduction and the growth of these cells were monitored using a real-time cell analysis (RTCA) system. The expression of the transgenes were verified by western blot and immunofluorescence (Figure 2E–2F and Supplementary Figure S1). As expected, eGFP-expressing HeLa or CaSki cells grew at a similar rate to the parental cells, indicating the lentivrial transduction and expression of the irrelevant eGFP gene had no apparent impacts on the growth of these cervical cancer cells. By contrast, hBD3 overexpression significantly enhanced the growth of both cell lines (Figure 2A–2B). This growth stimulating effect of hBD3 was associated with increased S phase population as determined by PI cell cycle analysis (Figure 2C–2D) and was accompanied by the induction of multiple cell cycle regulators including Cyclin A, D1/D2, E, CDK2 and CDK4, although the identities of the key regulators varied between the two cell lines (Figure 2E–2F). Together, our results demonstrated that hBD3 promotes the G1/S progression and thus the proliferation of cervical cancer cells.

### hBD3 does not protect cervical cancer cells against chemo-drug induced apoptosis

**Figure 3:**
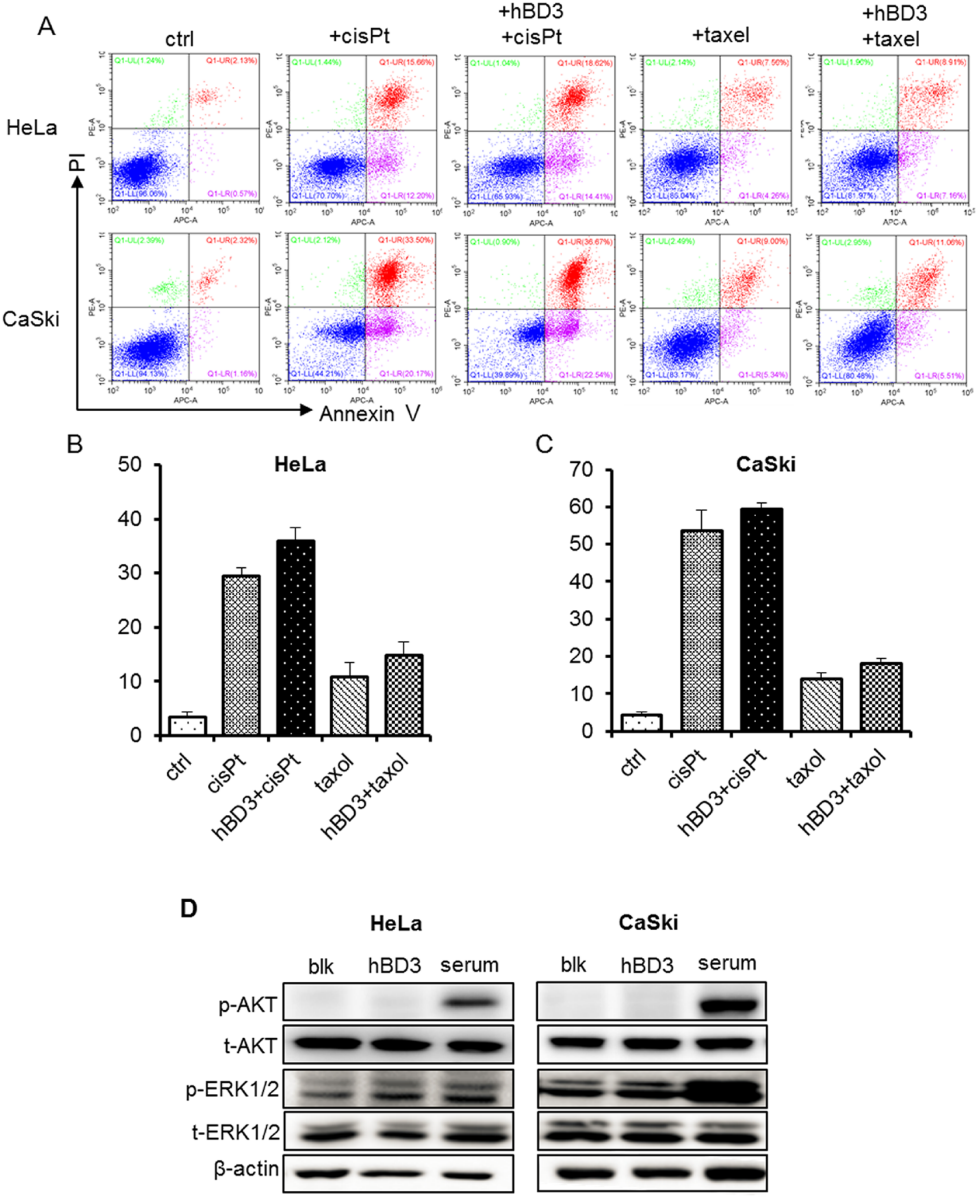
Effects of overexpression of hBD3 on apoptosis of HeLa and CaSki cells **A.** HeLa and CaSki cell apoptosis was analyzed by Annexin V/PI staining and flow cytometry after 48 hours of 40μM cis-platin and 500nM Paclitaxel treatment. **B, C.** Quantitation of the results from (A). **D.** Phosphorylation of ERK and AKT of parental and hBD3-overexpressing cervical cancer cells was analyzed by western blot with β-actin being an internal control. Serum stimulation was set as the positive control.

Previous studies have shown that hBD3 exhibited anti-apoptotic activity in cisplatin-treated SCCHN cells by activating the PI3K/AKT pathway [16]. To test whether hBD3 has similar protective effect in cervical cancer cells, we comparatively analyzed the apoptosis of parental cervical cancer cells (HeLa and CaSki) and their hBD3-expressing counterparts using AnnexinV/PI staining. Cells were treated with cisplatin or Paclitaxel to induce apoptosis since the basal apoptosis levels were low in these cell lines. As expected, cisplatin induced ~30% and ~50% apoptosis in HeLa and CaSki cells respectively, and Paclitaxel treatment led to around 10% cell death in both cell lines (Figure 3A–3C). In contrast with previous study on SCCHN cells, expression of hBD3 did not reduce chemo-drug induced cell death and even slightly enhanced it. Moreover, we did not observe activation of the anti-apoptotic effectors such as ERK and AKT in hBD3 treated cells, although serum, as a positive control did enhance the phosphorylation of both ERK and AKT (Figure 3D). Therefore, the anti-apoptotic effect of hBD3 against chemo-drugs is likely dependent on cellular context.

### hBD3 promotes the migration/invasion ability of cervical cancer cells

**Figure 4:**
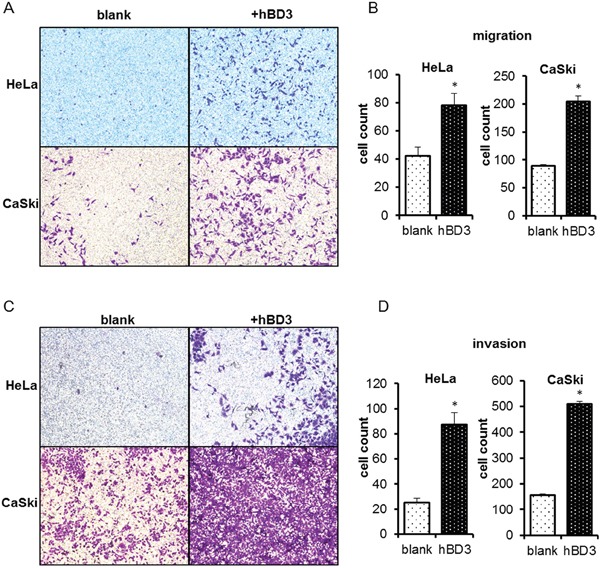
Effects of overexpression of hBD3 on migration/invasion ability of HeLa and CaSki cells Transwell assays were performed on HeLa and CaSki to measure cell migration **A.** or invasion **C.** Quantitation of results from (A) and (C) was shown in **B** and **D.** respectively. Quantitative data were presented as mean±SEM (n=6 for migration assay, n=5 for invasion assay), *P<0.05.

*In vitro* Transwell assays were performed to investigate the impact of hBD3 on the migration and invasion of HeLa and CaSki cells. Parental CaSki cells had stronger basal migration and invasion capacities than the parental HeLa cells. Nevertheless, hBD3-overexpression resulted in a two-fold increase in the migration of both cell lines and improved the invasion capacity by more than three-fold, indicating over-expression of hBD3 significantly increased the motility and invasiveness of both cell lines (Figure 4A-4D).

### hBD3 promotes the growth of cervical cancer cells by activating the NF-κB signaling

**Figure 5:**
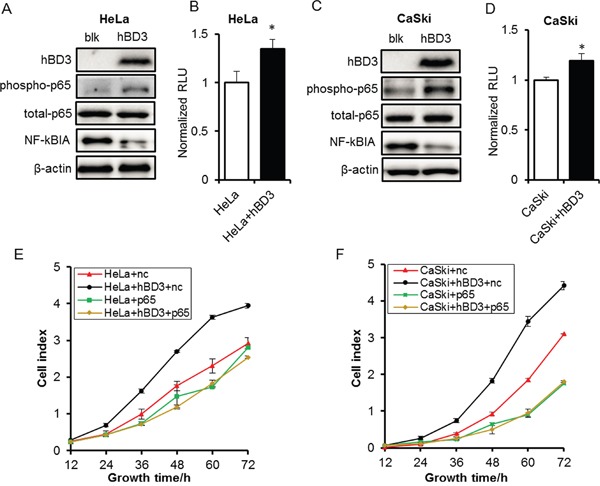
Effects of overexpression of hBD3 on NF-κB signaling The expressions of phospho-p65, total p65 and NF-κBIA were analyzed by western blot in HeLa **A.** and CaSki **C.** cells with or without overexpression of hBD3. β-actin was used as a loading control. Activity of NF-κB signaling of HeLa **B.** and CaSki **D.** analyzed by dual-luciferase assay. The luciferase readings were normalized accordingly and plotted against each treatment condition. Quantitative data were presented as mean±SEM (n=9), *P<0.05. **E-F.** Cell growth monitored by the xCELLigence RTCA system after p65-depleted in parental or hBD3-overexpressing HeLa and CaSki cells. Quantitative data were presented as mean±SEM (n=3), *P<0.05.

To understand the molecular mechanisms of the oncogenic effects of hBD3, we examined several key pathways that have been reported to be activated by hBD3 and implicated in the oncogenesis of different cancers [13, 16, 22]. The ERK and AKT signaling were not affected by hBD3 overexpression as clearly illustrated by the phospho-ERK and phospho-AKT western blots (Figure 3D). Of note, over-expression of hBD3 enhanced the phospho-p65 level without altering the total p65 expression, and reduced the level of the NF-κB inhibitor, IkBα, suggesting that the NF-κB pathway was more active in hBD3-expressing cells than in the parental cells (Figure 5A, 5C). Consistently, dual luciferase assay using the pLuc-NFκB reporter construct further confirmed that NF-κB activity was about 30% higher in hBD3-expressing cells than in parental cells (Figure 5B, 5D).

To test whether NF-κB is a downstream effector of the oncogenic function of hBD3, the NF-κB family member p65 was depleted using specific siRNAs and the impact on the growth of parental or hBD3-expressing cells was investigated (Figure 5E–5F). Knockdown of p65 mildly inhibited the growth of parental HeLa and CaSki cells, suggesting that NF-κB activity plays a contributive role in the growth of these cervical cancer cells. Consistent with prior observation, hBD3 over-expression dramatically enhanced the proliferation of control siRNA treated HeLa or CaSki cells. Importantly, p65 depletion completely abolished the growth-promoting effect of hBD3 and reduced the growth rate of hBD3-expressing cells to a level comparable to the p65-depleted parental cells (Figure 5E–5F, Supplementary Figure S2). Taken together, our results suggested that hBD3 contributes to the growth of cervical cancer cells at least partly by activating the NF-κB pathways.

### hBD3 promotes the tumor formation of cervical cancer cells *in vivo*

**Figure 6:**
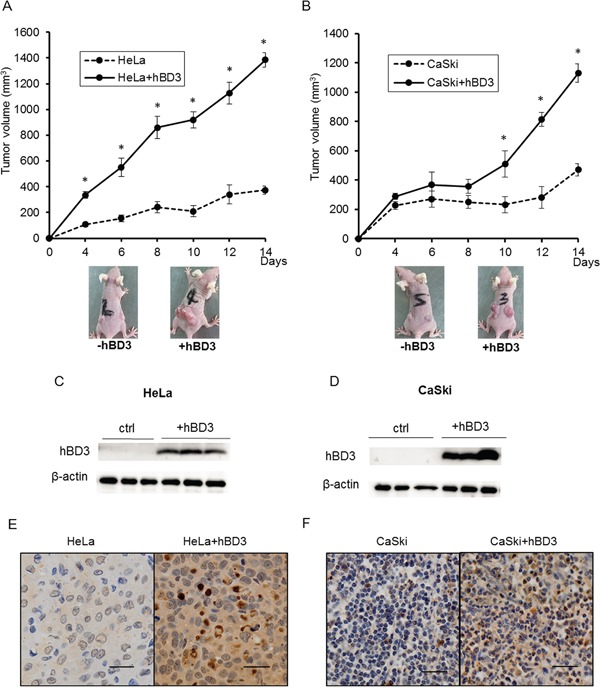
hBD3 overexpression promotes the growth of cervical cancer cells tumor xenograft *in vivo* The growth curve of the hBD3-overexpressing HeLa **A.** and CaSki **B.** xenograft in the nude mouse model. The graph shows the mean ± SEM (n=8); **P<0.05. **C, D.** Western blot analysis of hBD3 expression in the xenografts. β-actin is used as an internal control. **E, F.** Immunohistochemical analysis of the expression of Ki67 in the exnografts. scale bar=25μm.

To assess the effects of hBD3 expression on tumor formation *in vivo*, xenograft models of parental or hBD3-expressing HeLa and CaSki cells were established by implanting cells into left and right flanks of the nude mice and tumor growth was monitored over time. In line with the *in vitro* results, hBD3 overexpression significantly promoted the formation and the growth of tumors *in vivo*. By the end of the experiment (Day 14), tumors from the hBD3-overexpressing group have much bigger sizes than tumors from the control group (Figure 6A–6B). The expression of hBD3 at the end point of the experiment was confirmed by western blot (Figure 6C–6D). The expression of Ki67, a well-known cell proliferation marker, and activation of NF-κB were examined in the tumor xenografts tissues by immunohistochemical staining. As shown in Figure 6E-6F, the expression of Ki67 in the tumor tissues formed by the hBD3-overexpressing HeLa and CaSki cells was increased compared with the parental cells. Consistent with the *in vitro* data, enhanced phospho-p65 level was also observed in the hBD3-overexpressing xenografts (Supplementary Figure S3).

## DISCUSSION

An increasing amount of evidence has implicated the antimicrobial peptide, hBD3 in the carcinogenesis of head and neck cancers [12, 13, 16, 22], although the mechanism of its oncogenic function has not been fully understood. A recent study showed that hBD3 expression was regulated by the high-risk human papillomavirus E6 protein via p53 in oral squamous cell cancers [21]. This intriguing finding prompts the question whether hBD3 is expressed in another HPV-inducing cancer, cervical cancer and if so, whether hBD3 plays a role in the progression of the disease. In this study, we provided first evidence that hBD3 expression was elevated in cervical cancer samples and correlated with disease progression. Further studies showed that hBD3 enhanced cervical cancer cell proliferation and migration *in vitro* via activation of the NF-κB signaling and also promoted tumorgenesis *in vivo*.

Our IHC results showed that above 90% of the cervical cancer tissue samples stained positive for hBD3, supporting the presence of a correlation between hBD3 and the disease. Interestingly, a follow-up detection for HPV virus using a combinational probe set targeting three high-risk virus strains, HPV16, 18 and 58 revealed that all the cervical cancer samples were HPV positive (data not shown). Thus, it is likely that the expression of hBD3 in cervical cancer cells is also regulated by the HPV E6 protein.

The expression of hBD3 in epithelial cells or keratinocytes is known to be induced by various signals, such as virus, bacteria, pathogen-associated molecular patterns (PAMPs), or proinflammatory cytokines such as tumour-necrosis factor (TNF) and interleukin-1β (IL-1β) [23–26], mostly via an EGFR-dependent pathway [23, 27]. In human gingival epithelial cells, keratinocytes, and oral epithelial cells, it has been demonstrated that the presence of various Gram-negative and Gram-positive bacteria including *F. nucleatum*, *A. naeslundii*, *S. pyogenes* and their LPS or virulent factors could induce the release of hBD-3 and induce the production of inflammatory cytokines [5, 23, 28, 29]. To be noted, it has also been found that expression of hBD3 in the intestine was suppressed by *Shigella flexneri*, a highly contagious Gram-negative enteroinvasive bacteria that cause bacillary dysentery, which was considered as an example of targeted survival strategy of this enteropathogen [30]. Besides the bacterial stimuli, hBD3 upregulation has been found in response to viral infection including human immunodeficiency virus (HIV) infection [31], human rhinovirus infection [32] and HPV infection [26, 33, 34]. A previous study showed that hBD3 expression was moderately induced (2.5-fold) in clinically healthy tissues of HPV-infected women and much more dramatically induced (10-fold) in biopsies of HPV-infected genital warts, compared with normal tissue of uninfected women [33–35]. We speculated that HPV infection-induced hBD3 expression leads to the recruitment and activation of macrophages which release cytokines and chemokines to further induce hBD3 expression and foster a local inflammatory microenvironment, promoting tumor formation and progression. This model may explain why hBD3 expression appears to be much higher in tissue samples than in the cell lines, which apparently lacks the microenvironment.

The growth-stimulating effects of hBD3 was first reported by Jin et al. on HEK293 cells implanted in nude mice [17] and later confirmed by Winter et al. in oral cancer cells [12]. Consistent with prior findings, our results showed that hBD3 promoted the growth of cervical cancer cell lines. Cell cycle analysis revealed that hBD3 overexpression resulted in an increase in the S phase population and a concomitant induction of multiple G1/S regulators such as cylin A, D1/D2, E, CDK2 and CDK4. Tumor xenograft experiments further proved that hBD3 expression promoted tumor formation and growth *in vivo*. Thus, we provided consistent *in vitro* and *in vivo* data to support the growth-promoting role of hBD3 in cervical cancer. However, the molecular details of cell cycle regulation by hBD3 requires further investigation.

Mburu et al. reported that exogenous hBD3 could protect SCCHN cells against cisplatin-induced apoptosis by activating the PI3K/AKT pathway [22]. We did not observe this protective effect in cervical cancer cells and no activation of the AKT or ERK signaling was seen either. We suspected that the anti-apoptotic function of hBD3 may depend on cellular contexts.

The effect of hBD3 on the migration/invasion capacity of cervical cancer cells was also investigated in this study and we found that hBD3 promoted the migration/invasion of cervical cancer cells. Similar results were reported by Mburu et al showing hBD3 promoted migration of SCCHN cells toward CCR19, a ligand for the lymph node homing receptor CCR7, by upregulating the expression of CCR7 [22]. However, we did not observe increased CCR7 expression in cervical cancer cells by immunoflurosence staining (data not shown). By contrast, Uraki et al. reported an inhibitory effect of hBD3 on cell migration in colon cancer cells without exhibiting any growth-stimulating effects [38]. Therefore, hBD3 may exert its impact on cell migration/invasion through different mechanisms.

So far, the molecular mechanism for the oncogenic function of hBD3 remains unclear. hBD3 was found to activate oncogenic signalings such as ERK1/2, AKT and NF-κB in various cells [6, 13, 17, 22]. Although the ERK1/2 and AKT signaling was not affected by hBD3 in cervical cancer cells, the NF-κB pathway was clearly activated by overexpression of hBD3 (Figure 5). Thus, hBD3 may function through NF-κB to promote the oncogenesis in cervical cancer cells. Indeed, knockdown of the NF-κB component, p65 completely blocked the growth-stimulating effect of hBD3 (Figure 5E–5F). In addition to the intracellular signals, hBD3 may also exert its oncogenic function through the extracelluar microenvironment. Jin et al. showed that hBD3 expression in tumor microenvironment was associated with intratumoral accumulation of CCR2-expressing macrophages in oral CIS lesions and in nude mice model and that hBD3 induced production of multiple cytokines such as IL-1α, IL-6, IL-8 by macrophages [16, 17]. Thus, hBD3-recruited TAMs, together with the cytokines they produced may provide a tumor-promoting microenvirnment.

In conclusion, our study demonstrated that hBD3, by potentiating NF-κB activity, exhibits remarkable tumorigenic activities *in vitro* and *in vivo,* and therefore may serve as a new target or biomarker for early prevention, treatment and prognostic purpose.

## MATERIALS AND METHODS

### Patient samples

Patient samples were obtained from the tissue collection of Department of gynecology, the First Affiliated Hospital of Xi'an Jiaotong University. There were a total of 22 normal cervix samples, 41 carcinoma in situ (CIS) samples and 37 cervical cancer (SCC) samples without chemotherapy, immunotherapy, or radiotherapy. The differentiation stages of all samples were determined by two experienced pathologists. Informed consent for tissue procurement was obtained from all patients before study initiation, and Ethics approval was obtained from the Institutional Ethics Committee of School of Life Science and Technology, Xi'an Jiaotong University (Approval No. 2014011).

### Immunohistochemistry

Immunohistochemical staining was performed using a standard immunoperoxidase staining procedure [23]. The staining results were evaluated under microscope by two independent pathologists and quantitated based on the following scoring system. A positive rate score was first assigned according to the percentage of positive cells (≤5%, scored 0; 6%~25%, scored 1; 26%~50% scored 2; 51%~75% scored 3; >75%, scored 4). A second score of staining intensity was then assigned (colorless scored 0, light yellow scored 1, yellowish brown scored 2 and chocolate brown scored 3). The overall score for each microscopic field was calculated by the product of the two scores. The average score of five fields was taken as the final score of hBD3 expression for each slide.

### Cell culture

The human cervical cancer lines HeLa (ATCC-CCL2) and CaSki (ATCC-CRL-1150) was purchased from ATCC (USA). The cells were maintained in 1640 supplemented with 10% FBS, penicillin (100 U/ml), and streptomycin (100 μg/ml). Cells were routinely cultured in a humidified incubator at 37°C and 5% CO_2_.

### Lentivirus transduction

eGFP or hBD3-expressing lentiviral constructs were generated and lentiviral particles were produced in 293FT cells as previously described [36]. Transduction was carried out by adding 10μl of virus suspension (titer 1×10^9^ TU/ml) to cells cultured in l ml complete culture medium containing 5μg/ml polybrene. Forty eight hours after transfection, cells were selected with puromycin (5μg/ml) containing medium for two weeks.

### qRT-PCR

Total RNA was extracted from the cells using the Trizol reagent (Invitrogen, Carlsbad, CA), and then reverse transcribed into cDNA. The PCR primers were synthesized by Sangon Biotech (Shanghai, China). Primer sequences are shown in Supplementary Table S1. Reactions were performed using SYBR premix EX Taq I (Takara, Japan) and analyzed by the CFX Connect™ Real-Time PCR Detection System (Bio-Rad, USA). Relative mRNA levels were calculated using the comparative Ct (ΔCt) method. Experiments were repeated three times and three technical replicates were included for each data point.

### Western blot

Protein expression levels were analyzed by standard Western blotting protocols. The following antibodies were used in this study: hBD3 antibody (500-P241) from PeproTech (Rocky Hill, NJ, USA); AKT (pan) (#4691), ERK 1/2 (#9102), phospho-AKT (#2965), phospho-ERK1/2 (#4376), p65 (#8242) and phospho-p65 (#3033) antibodies from Cell Signaling Technology (Danvers, MA, USA). Cyclin A (sc-596), cyclin E (sc-247), Cdk2 (sc-163) and Cdk4 (sc-260) from Santa Cruz Biotechnology (Dallas, TX, USA); β-actin (SP124) antibody from Sigma-Aldrich (USA). Cyclin D1/D2 was from R&D Systems (Minneapolis, MN, USA).

### Cell proliferation assay

Real-time cell growth was assessed using the xCELLigence RTCA system (ACEA Biosciences, USA) according to the manufacturer instruction. Briefly, cells (parental or hBD3-expressing) were seeded at a density of 5×10^3^ cells/well in the gold-coated plates. Real-time growth of the cells were monitored and the growth data (cell index) were acquired every five minutes by the system [37]. Three technical replicates were performed and data were collected for a duration of 72 hours. Mean readings from three independent experiments were plotted for each chosen time point.

### PI cell cycle analysis

Cells were harvested, fixed in 70% ethanol, and stored overnight at −20°C. For the analysis, PI staining solution (50 μg/mL PI and 100 μg/mL RNase A) was added to the cells, and then incubated for 30min in the dark at 37°C. The cells were analyzed using flow cytometry (CytoFlex from Bechman Coulter, USA). Modifit software was used to analyze the results. Three independent experiments were performed.

### Annexin V/PI staining

Cells were treated with 40μM cisplatin or 500nM paclitaxel for 24h before harvest to induce apoptosis. Cells were then harvested and stained with the Annexin V-APC/PI Apoptosis Detection Kit (Biolegend) following the manufacturer's instructions. Stained cells were analyzed by flow cytometry (FACSCalibur from BD Biosciences, USA). Three independent experiments were performed.

### Transwell migration and invasion assay

For the migration assay, parental or hBD3-overexpressing cells were added to each Transwell chamber (8μm pore size, Millipore, USA) seated a in 24-well plate and 500 μl of culture medium with 10% FBS was added to the lower chamber. After culturing for18h (for CaSki cells) and 24h (for HeLa cells), the residual cells on the top surface of the membrane were gently wiped out with a cotton swab. The cells that migrated to the bottom surface of the membrane were stained with staining solution (0.1% crystal violet). Average number of migrated cells was obtained from countings of five microscopic fields (up, down, left, right and middle). Invasion assay was performed similarly except that the Transwell chamber membrane was pre-coated with Matrigel (BD Biosciences, USA) and the culture time was extended to 36h (for CaSki cells) and 48h (for HeLa cells). Migration and invasion assays were repeated three times and three technical replicates were performed for each data point.

### Promoter reporters and dual luciferase assays

HeLa and CaSki cells (with or without hBD3-overexpression) were transfected with pNFκB-luc plasmid (1 μg/33 mm well) using X-tremeGENE HP transfection reagent (Roche, USA). Transfection efficiency was normalized by cotransfection with a pRL-TK reporter containing a full-length renilla luciferase gene (Promega, Madison, WI, USA) as an internal control reporters. 72 hours after transfection, the cells were harvested in passive lysis buffer (Promega, Madison, WI, USA). Firefly luciferase and renilla luciferase activities were quantified using the dual luciferase assay system (Promega). Changes in luciferase activity were calculated relative to the luciferase activity of pRL-TK in HeLa and CaSki cells, which was given the reference value of 1 as described. The luciferase readings were normalized accordingly and plotted against each treatment condition.

### p65 siRNA knockdown

The p65 siRNA oligonucleotides were synthesized by Shanghai GenePharma (Shanghai, China) and their sequences are shown in Table S1. Cells were transfected with siRNAs according to the recommended procedures of Lipofectamine™2000 Transfection Reagent (Invitrogen, Carlsbad, CA).

### Animal studies

Female BALB/c/nu nude mice (3-4 weeks old) were purchased from Animal Center of Xi'an Jiaotong University. Five animals were individually inoculated subcutaneously on their left and right flanks with 5×10^6^ HeLa cells and hBD3-overexpressing HeLa cells respectively. The other five animals were similarly inoculated with CaSki cells and hBD3-overexpressing CaSki cells. Tumor sizes were measured every 2 days with a digital microcaliper, and tumor volumes were calculated using the following formula: tumor volume = length×width×width/2 (mm^3^). After 14 days, the mice were sacrificed and the tumors were excised for western blot and histological analysis. All animal protocols were approved by the Institutional Animal Care and Use Committee of Xi'an Jiaotong University.

### Statistical analysis

All data are presented as the Mean ± SEM. The SPSS statistical package (SPSS, Chicago, IL, USA) was used for data analysis. Pearson chi-square test and Wilcoxon signed-rank test were used for immunohistochemistry analysis. Student's t test was used for comparisons between two groups, and one-way ANOVA test was used for experiments with more than two groups. P < 0.05 was considered statistically significant.

## SUPPLEMENTARY FIGURES AND TABLES


