# Supplementary material for: Human beta-defensin 3 contributes to the carcinogenesis of cervical cancer via activation of NF-κB signaling
Source: Oncotarget. 2016 Oct 4;7(46):75902–13. doi: 10.18632/oncotarget.12426 (PMC5342786; doi:10.18632/oncotarget.12426)
Supplement: Supplementary file 1 [file oncotarget-07-75902-s001.pdf]

Human beta-defensin 3 contributes to the carcinogenesis of cervical cancer via activation of NF-κB signaling

SUPPLEMENTARY FIGURES AND TABLE

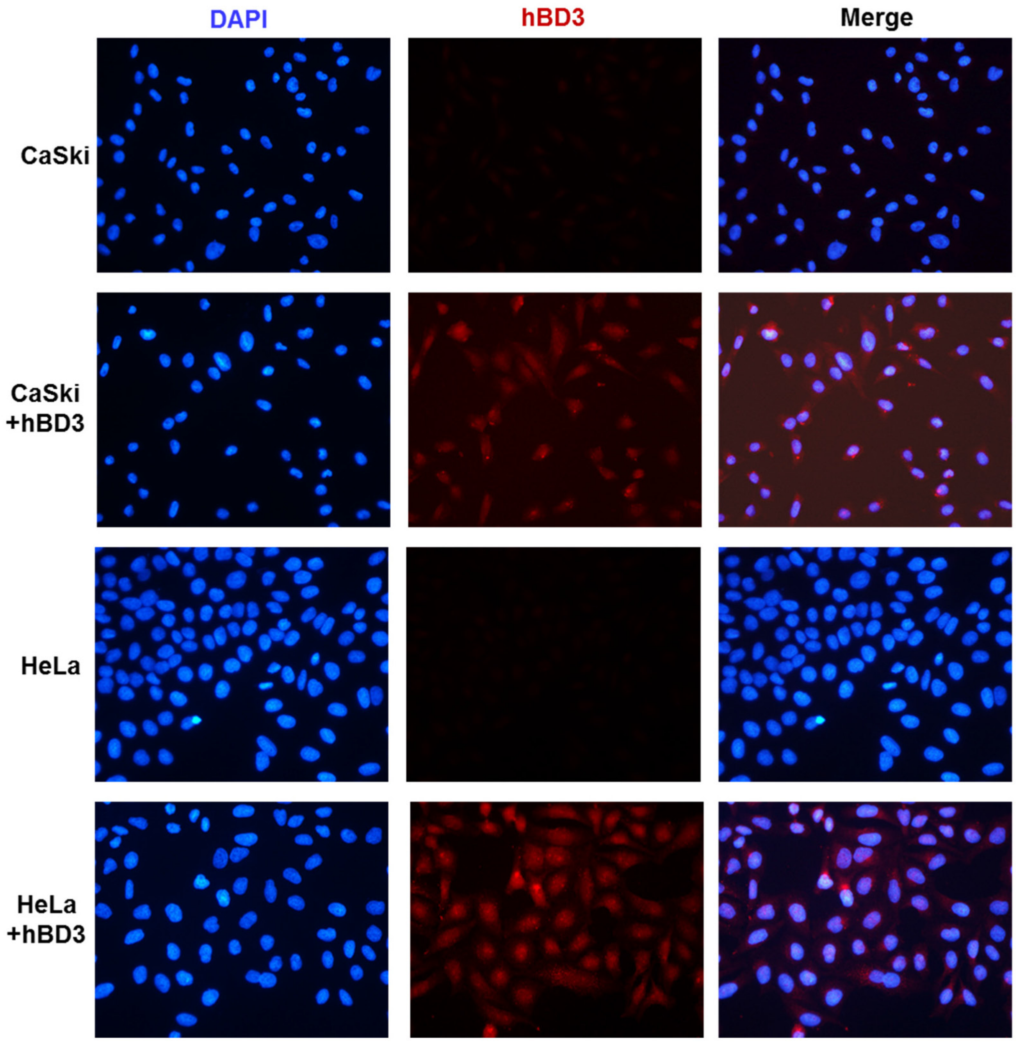

Supplementary Figure S1: Immunofluorescence analysis of hBD3 expression of parental and hBD3-overexpressing HeLa and CaSki cells.

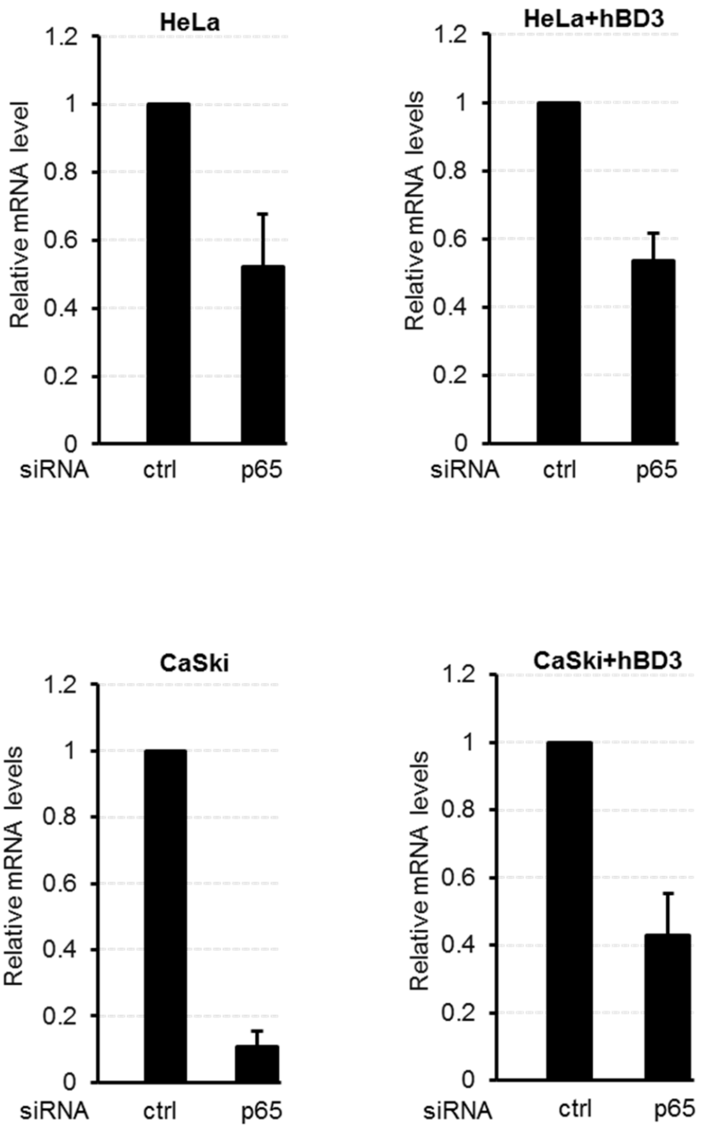

Supplementary Figure S2: The efficiency of p65 knockdown in parental and hBD3-overexpressing HeLa and CaSki cells.

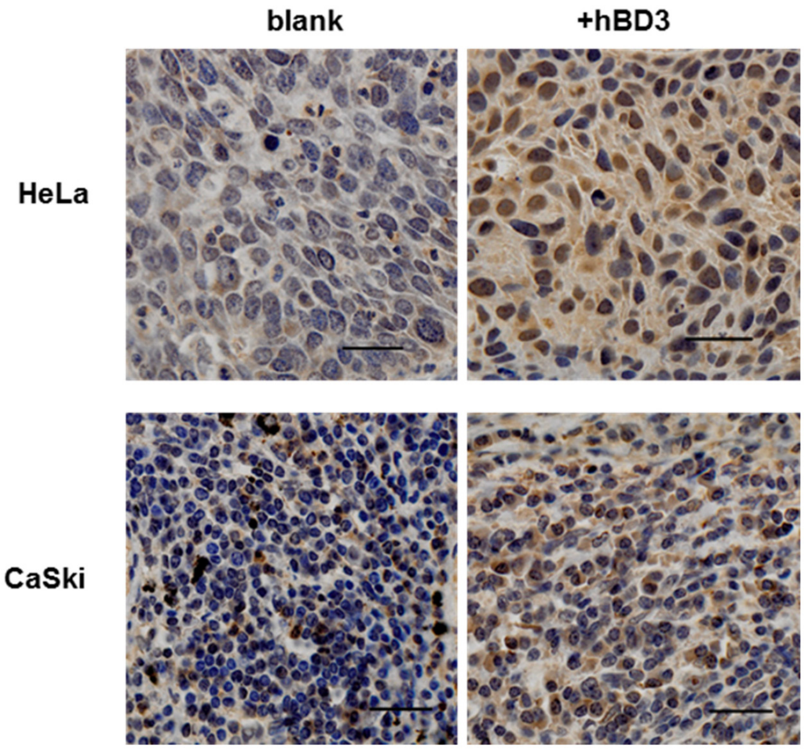

Supplementary Figure S3: The immunohistochemical analysis of phosph-p65 in the xenografts, scale bar=25µm.

Supplementary Table S1: The RT-PCR primers and sequences of siRNAs used in this study

Name	Target	Sequence
nc siRNA	Negative control	Forward : 5'-UUCUCCGAACGUGUCACGUTT-3'<br>Reverse: 5'-ACGUGACACGUUCGGAGAATT-3'
p65 siRNA	NF-κB p65	Forward: 5'-CCUCCUUUCAGGAGAUGAATT-3'<br>Reverse: 5'- UUCAUCUCCUGAAAGGAGGTT-3'
hBD3	RT-PCR	Forward: 5'-TGCTCTTCCTGTTTTTGGTGC-3'<br>Reverse: 5'-TGCCGATCTGTTCCTCCTTT-3'
p65	RT-PCR	Forward: 5'-GGGAAGGAACGCTGTCAGAG-3'<br>Reverse: 5'- TAGCCTCAGGGTACTCCATCA-3'
